# Genetic Parameters for Different Measures of Feed Efficiency and Their Relationship to Production Traits in Three Purebred Pigs

**DOI:** 10.3390/life11080830

**Published:** 2021-08-13

**Authors:** Kier Gumangan Santiago, Bryan Irvine Lopez, Sung-Hoon Kim, Dong-Hui Lee, Young-Gyu Cho, Yu-Na Song, Kang-Seok Seo

**Affiliations:** 1 Department of Animal Science and Technology, Sunchon National University, Suncheon 57922, Korea; 1190106@scnu.ac.kr (K.G.S.); a3832737@naver.com (D.-H.L.); dudrb1992@naver.com (Y.-G.C.); syn1010@naver.com (Y.-N.S.); 2 Department of Animal Science, Central Luzon State University, Science City of Muñoz 3120, Philippines; 3 Division of Animal Genomics and Bioinformatics, National Institute of Animal Science, Rural Development Administration, Iseo-myeon 55365, Korea; irvinelopez@korea.kr; 4 Pig Gene Korea Co., Ltd., Yongin 16866, Korea; drskim@naver.com

**Keywords:** genetic parameters, feed efficiency, residual feed intake

## Abstract

Residual feed intake (RFI) gained attention as a potential alternative to the feed conversion ratio (FCR). Thus, this study aimed to estimate genetic parameters for different feed efficiency (FE) traits (FCR, RFI1 to RFI5) and their genetic correlation to on-test daily weight gain (ADG), backfat (BFT), loin muscle area (LMA), lean percentage (LP), and total feed intake (FI) for 603 Male Duroc (DD), 295 Landrace (LL), and 341 Yorkshire (YY). The common spatial pen effect was also estimated in these traits. Five RFI measures were estimated by regressing daily feed intake on initial testing age (ITA), initial testing weight (IBW), and ADG for RFI1; other models were the same as RFI1 except for additional BFT for RFI2; LMA for RFI3; BFT and LMA for RFI4; BFT, LMA, and average metabolic body weight (AMBW) instead of IBW for RFI5. Genetic parameters estimated using two animal models and the REML method showed moderate heritability for FCR in all breeds (0.22 and 0.28 for DD, 0.31 and 0.39 for LL, 0.17 and 0.22 for YY), low heritability for the majority of RFI measures in DD (0.15 to 0.23) and YY (0.14 to 0.20) and moderate heritability for all RFI measures in LL (0.31 to 0.34). Pen variance explained 7% to 22% for FE and 0% to 9% for production traits’ phenotypic variance. The genetic correlation revealed that selection against less complex RFI1 in DD and LL and RFI2 in YY would bring the most advantageous reduction to FI (0.71 for DD, 0.49 for LL, 0.43 YY) without affecting ADG in all breeds (0.06 for DD, −0.11 for LL, 0.05 for YY), decrease in BFT, and increase in LP in DD (0.51 in BFT, −0.77 in LP) and LL (0.45 in BFT, −0.83 in LP). Therefore, inclusion of these breed-specific RFI measures in the future selection criteria would help improve feed efficiency in the swine industry.

## 1. Introduction

In the modern capital-intensive pork production system, the feed accounts for about 60% to 70% of the total cost of production [1]. Thus, any increase in the price of this input will pose limitations to the profitability of the livestock industry. At the same time, as the population increases, and there is a finite number of resources used for food production, competition between humans and livestock could be a potential problem in the future. Therefore, improving the feed efficiency (FE) of livestock and poultry would help prevent these foreseen problems. Azarpajouh et al. [2] also stated that improving FE could support industry competitiveness and reduce demand to global feed resources, which complement environmental sustainability. In the livestock and poultry industry, the FE is commonly measured through the feed conversion ratio (FCR) and gain to feed ratio (G:F), but less commonly measured using residual feed intake (RFI). The FCR and G:F ratio is obtained using two-component traits, particularly the feed intake and weight gain, while RFI is measured by subtracting actual daily feed intake (DFI) and predicted daily feed intake (pDFI). The pDFI can be obtained by regressing DFI to different production traits, carcass components, and maintenance requirements of the animal. Such difficulty in calculating RFI over ratio traits could probably the reason for the limited use of RFI in improving FE in the livestock and poultry industry. Given these various measurements of FE and differences in population structure, numerous researchers still obtained a low to moderate heritability for these FE traits, indicating their possible improvement once considered as part of the selection criteria. However, Gunsett [3] reported some problems in using ratio traits to improve FE, such as their unpredictable change in the future generation brought by the uneven selection pressure on their component traits. Such perceived problems in ratio traits gained the attention of various researchers on studying the potential of RFI as an alternative trait for ratio traits. In the study of Do et al. [4] and Lu et al. [5], it was found out that different measures of RFI have moderate to high heritability with weak to zero genetic and phenotypic relationships to on-test daily gain in weight (ADG) and backfat (BFT) and a high positive relationship to daily feed intake (DFI). According to Azarpajouh et al. [2], the pigs with low RFI consumed less feed than the predicted feed intake based on the production performance, and was therefore more feed efficient and economically sound in terms of lean production. Therefore, these findings suggest that the selection for pigs with low RFI would improve economic profitability of the swine industry by reducing feed intake without adversely affecting production traits, particularly ADG and BFT. Given these earlier claims for RFI, this study aimed (1) to estimate the genetic parameters, (co)variance components, and heritability estimates of different measurements of FE traits, (2) to estimate their genetic relationship to production and feed intake traits and, (3) to estimate the non-heritable common spatial pen effect for all studied traits in the Duroc, Landrace, and Yorkshire population used in this study.

## 2. Materials and Methods

### 2.1. Data

A total of 1239 male pigs at the growing-finishing phase comprising 603 Duroc (DD), 295 Landrace (LL), and 341 Yorkshire (YY) were used in the analyses. The DD pigs were born and reared in one GGP farm (Farm B), while LL and YY were both born and reared in two GGP farms (Farm A and Farm B) located in South Korea. Pigs from three pig breeds were born from the year 2018 to 2020. Each breeding farm was equipped with a different brand of single-space electronic feeders namely: the Schauer MLP II (Shauer Agrotonic GmbH, Prambachkirchen, Austria) and Acemo Genstar (Genstar, Acemo Skiold, Pontivy, France) for Farm A and Farm B, respectively. The number of animals for each pig breed and farm is shown in Table 1.

The pigs used in the study were initially reared until they moved to testing pens with an average initial testing age (ITA) and initial testing weight (IBW) of 82.99 ± 1.72 days and 43.71 ± 4.87 kg for DD, 79.49 ± 4.15 days and 36.93 ± 5.82 kg for LL, and 79.25 ± 4.57 days and 35.68 ± 6.76 kg for YY, respectively. Moreover, these tested pigs had free access to feed for an average of 62.33 ± 0.65 days for DD, 67.94 ± 7.23 days for LL, and 68.92 ± 6.88 days for YY. Approximately 9 to 12 pigs were contained by each testing pen equipped with an electronic feed dispenser.

### 2.2. Phenotypic Traits

Apart from feed intake records, other equally important production and feed efficiency traits were calculated and measured to facilitate the estimation of RFI, estimation of genetic parameters, and genetic and phenotypic correlation for all studied traits in this study. Among the calculated and measured traits are the following: average daily feed intake (ADFI), feed conversion ratio (FCR), on-test average daily gain (ADG), ultrasound backfat thickness (BFT), ultrasound loin muscle area (LMA), ultrasound lean percentage (LP) and average metabolic body weight (AMBW). The ADFI was calculated by dividing the total feed intake over the feeding days; the feed conversion ratio (FCR) was computed by dividing total feed consumed by each pig (kg) over the live weight gain (kg) during the experimental period; on-test average daily gain (ADG) during the experimental period was calculated by dividing weight gain or the difference of final body weight (FBW) and initial body weight (IBW) over the number of testing days.

The BFT was measured using A-mode scanners (PIGLOG 105, Frontmatec, Skive, Denmark) at three different sites (5 cm from the left and right of the midline), particularly on the shoulder or the 4th thoracic vertebrae, mid-back, or the last thoracic vertebrae, and pigs’ waist or at the last lumbar vertebrae. The BFT used in the analysis of RFI was not adjusted to 90 kg body weight; instead, actual measurements of BFT at off-test were averaged and included in the analyses. The LMA, which measures the largest muscles in pigs, was scanned at 5 cm ventral to the dorsal point of the last thoracic vertebrae using the same probe (PIGLOG 105) used to measure BFT. The same A mode scanner was also used to determine the LP in this study. Similar to BFT, the LMA and LP not adjusted to 90 kg body weight were used in the analyses.

The average metabolic body weight (AMBW) was calculated based on the equation presented by Noblet et al. [6] and NRC (2012) [7]. This calculated trait was similarly used in the study of Saintilan et al. [8] and Gilbert et al. [9]. The AMBW was calculated following the formula:

(1) $$\mathrm{AMBW}=\frac{\left({\mathrm{FBW}}^{1.60}-{\mathrm{IBW}}^{1.60}\right)}{1.60\times \left(\mathrm{FBW}-\mathrm{IBW}\right)}$$

where IBW is the initial testing weight; FBW is the final testing weight, and 1.60 which was constant in this equation.

### 2.3. Estimation of Residual Feed Intake

The RFI was estimated by subtracting the actual ADFI from the predicted DFI (pDFI). The pDFI was obtained by regressing ADFI to production, carcass components, and maintenance requirements. The PROC GLM of SAS package PC version 9.4 [10] was used to estimate the pDFI from five regression models. Furthermore, each model includes initial testing age (ITA) as the common fixed covariates, while only models 1 to 4 include initial testing weight (IBW) as their common fixed covariates. Moreover, each model includes a different combination of covariate traits related to pig production and maintenance requirements. Specifically, model 1 includes ADG; model 2 includes ADG and BFT; model 3 includes ADG and LMA; model 4 includes ADG, BFT, and LMA; and model 5 includes ADG, BFT, LMA, and AMBW. The IBW was dropped in the last model to avoid multicollinearity problems due to the high correlation between IBW and AMBW. The RFI1, RFI2, RFI3, RFI4 and RFI5 in this study were obtained by subtracting ADFI to estimated pDFI obtained using five regression models presented below:

pDFI1 = β_0_ + CG*_j_* + β_1_ITA*_i_* + β_2_IBW*_i_* + β_3_ADG*_i_* + e*_i_* (2)

pDFI2 = β_0_ + CG*_j_* + β_1_ITA*_i_* + β_2_IBW*_i_* + β_3_ADG*_i_* + β_4_BFT*_i_* + e*_i_* (3)

pDFI3 = β_0_ + CG*_j_* + β_1_ITA*_i_* + β_2_IBW*_i_* + β_3_ADG*_i_* + β_5_LMA*_i_* + e*_i_* (4)

pDFI4 = β_0_ + CG*_j_* + β_1_ITA*_i_* + β_2_IBW*_i_* + β_3_ADG*_i_* + β_4_BFT*_i_* + β_5_LMA_i_ + e*_i_* (5)

pDFI5 = β_0_ + CG*_j_* + β_1_ITA*_i_* + β_3_ADG*_i_* + β_4_BFT*_i_* + β_5_LMA*_i_* + β_6a_AMBW + e*_i_* (6)

where CG is the contemporary group of the pigs (testing batch or farm-testing batch) to account for the differences in managerial and environmental effects for each testing batch; β_0_ indicates the intercept; β_1_, β_2_, β_3_, β_4_, β_5_, and β_6_ represent the partial regression coefficients for ITA, IBW, ADG, BFT, LMA, and AMBW, respectively; e represents the residual term from the linear model above. The root mean square error (RMSE), coefficient of determination (R^2^), and Akaike information criterion (AIC) are the parameters used in determining the best regression model in predicting DFI for each breed. The AIC value was computed using the PROC GLMSELECT of the SAS package PC version 9.4 [10].

### 2.4. Statistical Analyses

Before analyses of genetic parameters, the genetic and phenotypic correlation, the contemporary group (CG, testing batch = 22 levels for DD, farm-testing batch = 36 levels for LL and 34 levels for YY), the parity of dam, and potential covariates for each trait including ITA and IBW for FCR and ADG, and the final testing age (FTA) and final testing weight (FBW) for BFT, LMA, and LP were tested for significance using the PROC GLM procedure of SAS package/PC version 9.4 [10]. After the initial analysis, only CG was found significant (*p* < 0.001) as a fixed effect, while IBW and FBW were found significant as fixed covariates for corresponding test traits described earlier. The fixed covariates used for each trait are presented in Table 2. Meanwhile, the CG and fixed covariates were found not significant for all RFI values. This finding could be attributed to the fact that CG, ITA, and IBW were included in the computation of RFI. Despite the non-significance of CG for RFI, it was still included as a fixed effect in the estimation of genetic parameters for RFI, in accordance with the studies of Gilbert et al. [9], Saintilan et al. [8], and Lu et al. [5].

The genetic parameters and (co)variance components for each feed efficiency and production trait per breed were estimated using two animal models with different random effects analyzed through the restricted maximum likelihood method (REML) of the WOMBAT program [11]. Animal models 1 and 2 were applied for DD:

(7) $$\mathrm{Model}\;1:\;y_{t}=\mathrm{Xb}+Z_{1a}+Z_{2c}+e$$

(8) $$\mathrm{Model}\;2:\;y_{t}=\mathrm{Xb}+Z_{1a}+Z_{2c}+Z_{3p}+e$$

while models 3 and 4 were applied for LL and YY:

(9) $$\mathrm{Model}\;3:{\;y}_{t}=\mathrm{Xb}+Z_{1a}+e$$

(10) $$\mathrm{Model}\;4:{\;y}_{t}=\mathrm{Xb}+Z_{1a}+Z_{3p}+e$$

where y_t_ is the vector of observations for each feed efficiency and production trait of DD, LL, or YY; b is the vector of fixed effects of CG (testing batch for DD and testing farm- batch for LL and YY); a is the vector of additive genetic effects; c is the vector for the common litter effect which was only applied for DD pigs due to the small difference between animals and number of litters for LL and YY; p is the vector for random common spatial pen effects; e is the vector for random residual effects. The animal genetic, common litter, and spatial pen terms were assumed to be independent and distributed as a ~ $N\left(0,A\sigma_{a}^{2}\right)$, c ~ $N\left(0,I\sigma_{c}^{2}\right)$, p ~ $N\left(0,I\sigma_{p}^{2}\right)$, and e ~ $N\left(0,I\sigma_{e}^{2}\right)$, where $\sigma_{a}^{2}$, $\sigma_{c}^{2}$, $\sigma_{p}^{2},$ and $\sigma_{e}^{2}$ are variances of random animal genetic effect, random common litter effect, random common spatial pen effect, and random residual error effect, respectively; A is the numerator relationship matrix among animals; and I is the identity matrix. The X, Z_1_, Z_2_, and Z_3_ are the corresponding indices matrices for the fixed effect, additive genetic effect, common litter effect, and common spatial pen effect, respectively. The number of litters and animals in the pedigree data is presented in Table S1.

The heritability estimate (h^2^) was defined as the ratio of additive genetic $\left(\sigma_{a}^{2}\right)$ to phenotypic $\left(\sigma_{p}^{2}\right)$ variance as presented in the equation below:

(11) $$h^{2}=\frac{\sigma_{a}^{2}}{\sigma_{p}^{2}}$$

Bivariate analyses were performed to estimate the genetic and phenotypic correlation between feed efficiency and production traits. The first animal model for each breed including the fixed covariates for each trait was used for the said analyses. The estimated genetic correlation (r_g_) was calculated as:

(12) $$r_{g}=\frac{\sigma_{a12}}{\sqrt{\sigma_{a1}^{2}\times \sigma_{a2}^{2}}}$$

while the phenotypic correlation (r_p_) for each breed was calculated as:

(13) $$r_{p1}=\frac{\left(\sigma_{a12}+\sigma_{c12}+\sigma_{e12}\right)}{{\left[\left(\sigma_{a1}^{2}+\sigma_{c1}^{2}+\sigma_{e1}^{2}\right)\left(\sigma_{a2}^{2}+\sigma_{c2}^{2}+\sigma_{e2}^{2}\right)\right]}^{1/2}}$$

(14) $$r_{p2}=\frac{\left(\sigma_{a12}+\sigma_{e12}\right)}{{\left[\left(\sigma_{a1}^{2}+\sigma_{e1}^{2}\right)\left(\sigma_{a2}^{2}+\sigma_{e2}^{2}\right)\right]}^{1/2}}$$

where r_g_ is applied in all breeds; r_p1_ is applied in DD; and r_p2_ is applied for both LL and YY; $\sigma_{a12}$ is the genetic covariance between trait 1 and trait 2; $\sigma_{c12}$ is the common litter covariance between trait 1 and trait 2; $\sigma_{e12}$ is the residual covariance between trait 1 and trait 2; $\sigma_{a1}^{2}$ is the genetic variance of trait 1; $\sigma_{a2}^{2}$ is the genetic variance of trait 2; $\sigma_{c1}^{2}$ is the common litter variance of trait 1; $\sigma_{c2}^{2}$ is the common litter variance of trait 2; $\sigma_{e1}^{2}$ is the residual variance of trait 1; and $\sigma_{e2}^{2}$ is the residual variance of trait 2.

### 2.5. Model Comparison

To compare two models with a different random effect, the obtained Akaike information criterion [12] or AIC score for each model was used as the basis of comparison. The AIC was used to compare models with similar fixed effects but with different variance structures. The AIC score was obtained using the equation:

(15) $$\mathrm{AIC}=-2\log({\mathrm{ML}}_{k})+2p_{k}$$

where ML_k_, indicates the maximum log-likelihood for model k; p_k_, number of parameters for model k. The model with the lowest AIC score is identified as the best model fit. Moreover, the model with an absolute AIC score difference (Diff) less than the cut-off threshold of ≤2 is considered essentially as good as the best model fit [13], while the model with Diff ≤6 should not be discounted [14].

## 3. Results and Discussion

### 3.1. Residual Feed Intake (RFI)

The partial regression coefficients for each covariate used in computing the predicted feed intake are presented in Table 3. As observed in the results of our analyses, there is a small difference in terms of the R^2^ value between models used in predicting DFI. The phenotypic variability of ADFI due to RFI ranged from 22.82% to 23.72% in DD, 16.46% to 19.53% in LL, and 17.67% to 20.17% in YY. The contemporary group accounts for differences in the environmental and managerial aspect for each testing batch and testing farm-batch; fixed covariates such as ITA and IBW account for differences in the initial testing age and weight among animals, respectively; ADG represents the growth performance of pigs during the testing period; BFT, LMA, and LP represent carcass components; and AMBW represents the maintenance requirements of each pig. All of these fixed effects and covariates accounted for the variability in ADFI of approximately 76.28% to 77.18% in DD, 80.47% to 83.54% in LL, and 79.83% to 82.33% in YY. In agreement with the studies of Johnson et al. [15] and Hoque et al. [16], the regression models with BFT as a covariate had the highest R^2^ value indicating the importance of including BFT in predicting the DFI of pigs. Among production, carcass components, and maintenance requirement traits included in the RFI models used in this study, only ADG, BFT, and AMBW were found significant, while LMA was not significant but remains worthy to be included in the model to determine its impact in the prediction accuracy.

The observed R^2^ in this study was similar to that observed by Rauw et al. [17] with an overall 81% coefficient of determination obtained using the multiple linear regression of feed intake on metabolic body weight (MBW^0.75^), body weight gain, BFT, and ITA. Moreover, the same researchers stated that a higher R^2^ value could be attributed to the computation of the RFI value in pigs of same-sex, breed, and birth year. Meanwhile, the high R^2^ value in this study could also be associated with the report of Rauw et al. [17] with the difference that pigs tested in this study were born within a three-year period. Another reason for such a high R^2^ could be due to the inclusion of the testing batch and testing farm-batch as a fixed effect in predicting DFI.

However, previous researchers reported lower R^2^ values. Hoque et al. [16] reported a lower R^2^ of 59%, 68%, 61%, and 69% for RFI1, RFI2, RFI3, and RFI4 of DD pigs, respectively. Although this study used similar covariates for RFI1 to RFI4 with that of Hoque et al. [16], the observed difference in R^2^ might be due to the differences such as population structure, differences in fixed effects used to account for the environmental and managerial differences during the particular testing period, variations in initial testing age and weight, and nutritional content of diets fed to the animals. In addition, de Haer et al. [18] identified factors that could bring variations in RFI values obtained using predicted feed intake models with metabolic body weight, weight gain, and carcass components as covariates. These factors are the differences in diet digestibility, variations in the absorption and use of absorbed energy and nutrient allotted for physical activity, temperature regulation, maintenance of body tissues, health condition, protein and fat net efficiency, and basal metabolic rate.

Despite differences in R^2^, the partial regression coefficient observed for each covariate in three purebred pigs had an almost similar magnitude to those observed by Hoque et al. [16], with the exception that some covariates differ in terms of direction. In DD and YY, a negative and positive regression coefficient was observed for ITA and IBW, respectively, while the opposite direction was observed for the regression coefficient of ITA in LL. The negative regression coefficient for ITA indicates that delayed starting age results in reduced DFI, while the positive coefficient for IBW indicates that heavier initial testing weight resulted in higher DFI [16,19].

Meanwhile, the partial regression coefficient for ADG ranged from 1.52 to 2.04 for DD, 1.33 to 2.09 for LL, and 1.28 to 2.00 for YY. These regression coefficients for ADG were within almost the similar range to those observed by Hoque et al. [16] and Mrode and Kennedy [19] for models with similar covariates in their study. The lowest value for the regression coefficient of ADG was found in RFI5 which could be associated with the inclusion of AMBW and exclusion of IBW.

### 3.2. Descriptive Statistics of Studied Traits

The means and standard deviation (SD) for the analyzed feed efficiency and production traits are presented in Table 4. The lowest mean ADFI, ADG, AMBW, and BFT of 2.11 kg/day, 900 g/day, 12.32 kg^0.60^, and 11.73 mm, respectively, were observed in YY. On the other hand, the same pig breed recorded the highest mean LMA and LP of 28.31 cm^2^ and 58.91%, respectively. This observed leanness or lower BFT and lower daily gain of YY compared to LL and DD were also observed by Mrode and Kennedy [19] who recorded breed differences of 0.652 mm and 0.787 mm in BFT, and 0.0216 kg/day and 0.0342 kg/day in ADG between LL and YY, and DD and YY, respectively.

Meanwhile, among the three pig breeds, the LL had an intermediate mean for ADFI, ADG, AMBW, BFT, LMA, and LP with their corresponding values of 2.14 kg/day, 905.63 g/day, 12.40 kg^0.60^, 11.96 mm, 27.80 cm^2^, and 58.24%, respectively, while the poorest mean FCR was also found in this breed with 2.37 kg feed/kg gain. Most of these observations were also observed in the study of Mrode and Kennedy [19], where LL had intermediate ADG and the poorest FCR compared to DD and YY breeds. Furthermore, Do et al. [4] also reported that LL had the poorest FCR when compared to DD and YY with breed differences of 0.05 and 0.07, respectively.

Among breeds, the DD had the highest mean value for ADFI, ADG, BFT, and AMBW, and the lowest for FCR with corresponding values of 2.48 kg/day, 1067.32 g/day, 14.23 mm, 13.43 kg^0.60^, and 2.30 kg feed/kg gain, respectively. On the other hand, the same pig breed also recorded the lowest LMA and LP of 26.03 cm^2^ and 56.95%, respectively. Similar to these observations, Mrode and Kennedy [19] reported that DD had the fastest growth rate, lowest FCR, and thickest BFT compared to LL and YY. Similar to the study of Do et al. [4], ADG was found highest for terminal sires or DD compared to LL and YY. In general, these observed breed differences may not always be the case due to various factors that could affect the value of production traits such as differences in initial testing age and weight, final testing age and weight, testing duration, nutrition, and environmental conditions.

**Figure 1:**
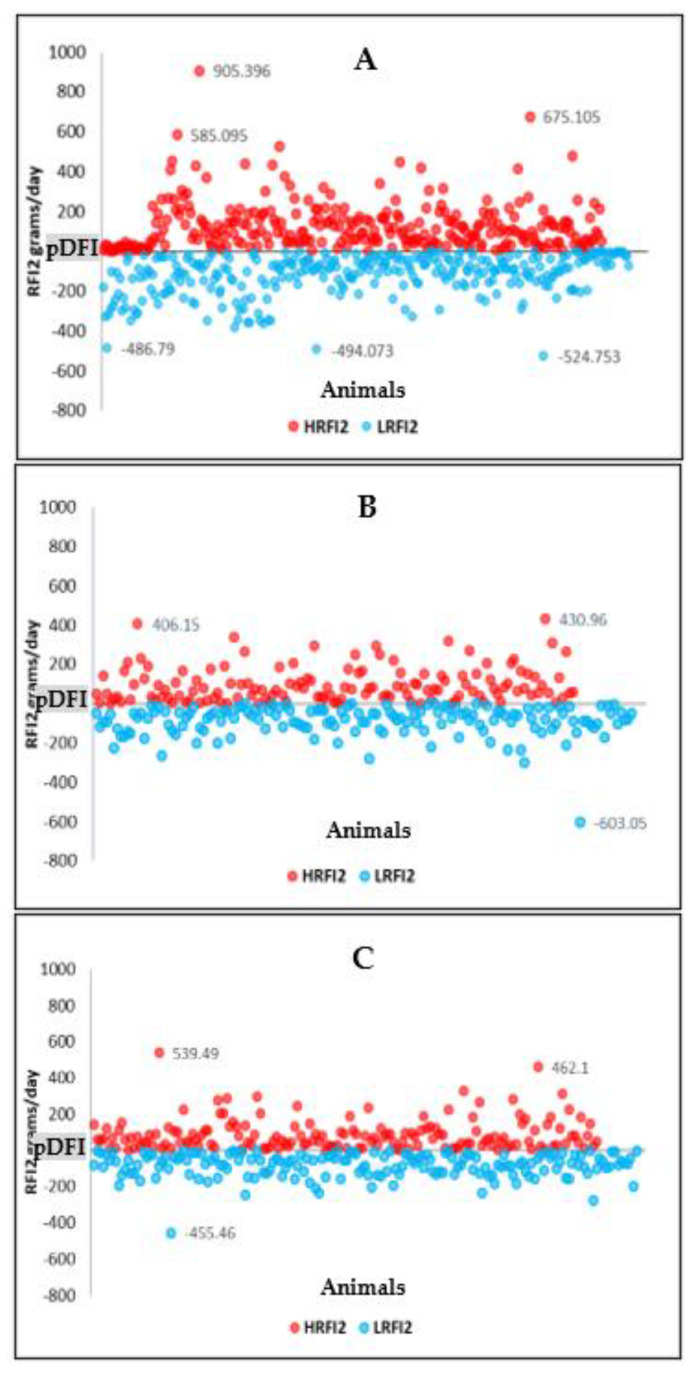
Duroc (**A**), Landrace (**B**), and Yorkshire (**C**) pigs with high residual feed intake (HRFI2) and low residual feed intake (LFRI2) based on the predicted feed intake (pDFI) obtained using RFI model 2 (RFI2).

The RFI values for all breeds in this study had the same value of zero as expected based on its definition. However, although the same mean value was observed, it was noticeable that each RFI model for each breed had considerable differences in the actual value as observed in their standard deviation (SD). The DD had the largest SD value ranging from 164.56 to 167.78 g/day, while YY had the lowest ranging from 113.92 to 121.70 g/day. Among RFI models, RFI2 had the best model in predicting feed intake in all breeds by having the lowest root mean square error (RMSE) and AIC scores (Table 3). Figure 1A–C, present the pigs with high RFI and low RFI against pDFI based on RFI model 2 (RFI2), where pigs with high RFI2 are classified as less feed efficient, while low RFI2 are more feed efficient as they eat more than and less than the pDFI, respectively.

The previously observed breed differences in RFI are in agreement with those observed by Do et al. [4] in DD having the highest SD for RFI ranging from 310 g/day to 320 g/day and YY having the lowest SD ranging from 270 g/day to 280 g/day. Similarly, Hoque et al. [20] reported higher SD for RFI in DD pigs ranging from 150 g/day to 160 g/day compared with only 50 g/day to 60 g/day in LL pigs. Both supporting findings used nearly similar covariates with the RFI models 1 and 2 in this study except that the fixed effect of the contemporary group and ITA (only by earlier research) was not included in their RFI analyses. Given the differences in the RFI models, these observations still indicate that DD had a wider range and possibly higher RFI value than other breeds.

### 3.3. Heritability Estimates of Feed Efficiency Traits

The heritability of feed efficiency and production traits of three pig breeds are shown in Table 5, Table 6 and Table 7. Estimates of heritability for FCR in this study were low to moderate ranging from 0.17 to 0.39. Different heritability estimates for FCR were observed in two models applied in three purebred pigs. Among animal models, model 1 had the highest heritability for FCR in all breeds. Given the differences in the number of additional random effects, the LL had the highest heritability estimates for FCR among breeds with an estimate of 0.31 and 0.39. Meanwhile, the heritability estimates for FCR in DD and YY were 0.22 and 0.28, and 0.17 and 0.22, respectively.

The observed heritability for FCR in LL was in agreement with those observed in the study of Hoque et al. [20], Do et al. [4], and Saintilan et al. [8] ranging from 0.31 to 0.35 but found higher than that observed by Labroue et al. [21] of 0.20. Meanwhile, the heritability estimates for FCR in DD with an additional random litter effect was in agreement with that obtained by Hoque et al. [22] of 0.27 but slightly lower than those observed by Do et al. [4] and Hoque et al. [20] of 0.30 and 0.31, respectively. Furthermore, the obtained heritability estimates for FCR in YY in this study were in agreement with that observed by Labroue et al. [21] with an estimate of 0.19 in the LW breed but were lower than those observed by Do et al. [4] of 0.32 in YY, Saintilan et al. [8] of 0.30 in the Large White (LW) sire and dam breed, and Kavlak and Uimari [23] of 0.28 in YY.

The estimated heritability for all RFI measures in this study was found to vary for each breed. In DD and YY, a low to moderate heritability estimates were observed in all RFI measures. Meanwhile, moderate heritability estimates were observed for all RFI measures in LL pigs. In all breeds, animal model 1 obtained the highest heritability estimates for all RFI measures due to the substantial pen effect for these traits. Among three breeds, the highest heritability was found in LL ranging from 0.30 to 0.34, where RFI measures with BFT as a covariate had the lowest heritability among all RFI measures in this breed. In DD, all RFI measures had low to moderate heritability estimates ranging from 0.15 to 0.23. Similar to LL, the DD also recorded the lowest heritability estimates in all RFI measures with BFT as a covariate (RFI2, RFI4, and RFI5). Among breeds, YY had the lowest heritability for RFI ranging from 0.14 to 0.20. Contrary to the earlier observations, the RFI measures for this breed that include BFT as a covariate had one to two points higher heritability than models without such covariate.

The heritability estimates observed for RFI in LL was in agreement with those observed by Do et al. [4] of 0.34 to 0.36 using two RFI models with almost similar covariates to RFI models 1 and 2 in this study, except that these researchers did not include CG as a fixed effect and ITA as a fixed covariate. Moreover, the observed heritability for RFI2 in this breed was in line with that indicated by Hoque et al. [20] for the RFI model with similar covariates having a heritability of 0.29 in LL pigs. Conversely, similar researchers also reported higher estimates of 0.47 for the same pig breed using the RFI model similar to RFI1 in this study with ITA, IBW, and ADG as covariates.

In DD pigs, the observed heritability for RFI2 and RFI4 was almost similar to those observed by Hoque et al. [16] for RFI models with identical covariates having an estimate of 0.22 and 0.20, respectively. However, the same authors also reported higher heritability for the model without BFT as a covariate (RFI1 and RFI3) with corresponding estimates of 0.38 and 0.33, respectively. Furthermore, Do et al. [4] likewise observed higher heritability estimates of 0.34 and 0.38 in RFI measures obtained using RFI models almost similar to RFI1 and RFI2 in this study. Such higher heritability for RFI measures without BFT as a covariate was also observed by Hoque et al. [20] in DD and LL, and Hoque et al. [16] in DD. Contrary to this, Do et al. [4] reported higher heritability estimates for RFI measures with BFT as a covariate.

The estimate of heritability for RFI in YY was similarly low as reported in the study of Gilbert et al. [9] of 0.14 in LW using RFI models with nearly similar covariates to RFI2 in this study. Furthermore, Johnson et al. [15] observed similar low heritability estimates (0.17, 0.11, 0.15, 0.10) for four RFI measures calculated in individually housed LW pigs using RFI models with similar covariates in this study. However, in a similar study by Gilbert et al. [9], a higher heritability of 0.24 was observed for the RFI model with covariates including ADG, lean meat content (LMC, %), and AMBW. Moreover, higher heritability estimates were observed in three different RFI models of Nguyen et al. [24] for LW, two different RFI models of Do et al. [4], and one RFI model of Kavlak and Uimari [23] for YY with corresponding estimates of 0.22 to 0.24, 0.39 and 0.40, and 0.32, respectively.

These observed differences in heritability estimates of feed efficiency traits, particularly RFI1, RFI2, RFI3, RFI4, and RFI5, could be attributed to differences in the number and sex of the tested animals, variations in the calculation of RFI such as the inclusion of a fixed CG to account for the environmental and management differences, inclusion of ITA and IBW when these traits are not fixed or with a large discrepancy among tested animals, variations in RFI as stated by de Haer et al. [18], and differences in pedigree information and fixed effects and covariates included in the animal models. Overall, the low to moderate heritability estimates obtained for all RFI measures in this study indicate a slow to moderate response of these traits to selection. Despite the lower heritability of RFI, this trait remains important especially when their genetic correlation to production traits and feed intake is considered.

### 3.4. Heritability Estimates of Production Traits

The heritability estimates for ADG observed in three pig breeds in this study ranged from 0.16 to 0.38. In ADG of DD and LL, animal model 1 had higher heritability compared to model 2, while identical heritability for both models was observed in ADG of YY (Table 8). The highest heritability for ADG (0.35 and 0.38) was observed in LL. Meanwhile, YY had an intermediate heritability estimate of 0.32. Among three pig breeds in this study, the DD had the lowest heritability for ADG with an estimate of 0.17 and 0.16 for animal models 1 and 2, respectively. These observed orders of breed based on the level of heritability of ADG was in accordance with the study of Do et al. [4] but not within a similar magnitude due to the higher heritability observed in their study with an estimate of 0.32 in DD, 0.54 in LL, and 0.47 in YY. The observed heritability for ADG of LL was similar with that of the study of Labroue et al. [21] of 0.41 for the same pig breed. Contrastingly, lower heritability for ADG was observed in the study of Saintilan et al. [8] of 0.26 in the French LL dam breed, while higher heritability was reported by Hoque et al. [20] and Chang et al. [25] of 0.47 and 0.49 in LL, respectively.

The heritability estimate for ADG in YY was in accordance with those observed by Gilbert et al. [9] of 0.35 in LW boars and 0.37 in LW females and castrated males and Saintilan et al. [8] of 0.33 in the LW dam breed. However, the latter researchers also reported a not different to zero heritability of 0.05 in the LW sire breed. Moreover, a lower heritability of 0.24 and 0.25 for ADG was reported by Johnson et al. [15] in LW and Kavlak and Uimari [23] in YY, respectively. Meanwhile, the observed heritability in ADG of DD was lower than those observed by Hoque et al. [16], Do et al. [4], and Lu et al. [5] with an estimate of 0.48, 0.32, and 0.23 to 0.35, respectively.

Estimates of heritability for BFT in this study were all high, ranging from 0.55 to 0.69. Both animal models 1 and 2 for each breed had obtained almost identical heritability due to the zero to very small common litter (for DD) and spatial pen effect for this trait. Among breeds, both DD and YY pigs recorded the highest heritability for BFT ranging from 0.68 to 0.69, while the lowest heritability of 0.55 (identical for models 1 and 2) was observed in LL. These observations are in agreement with several previously published research projects. Specifically, a high level of heritability in BFT was observed in the study of Cai et al. [26] of 0.68 in YY; Do et al. [4] in DD (0.54), YY (0.63), and LL (0.67); Hoque et al. [20] in DD (0.50) and LL (0.54); Saintilan et al. [8] in the French LL dam breed (0.61), LW dam breed (0.51), and LW sire breed (0.53).

A low to moderate heritability estimate for LMA was observed in this study ranging from 0.11 to 0.38. Due to a zero to very small fraction of the common spatial pen effect for this trait, almost identical heritability estimates were observed in both animal models 1 and 2 used in this study. The highest heritability for LMA was observed in LL with an estimate of 0.38 for both models 1 and 2. Meanwhile, intermediate heritability estimates of 0.18 and 0.19 for LMA were observed in YY, and the lowest heritability for LMA of 0.11 was observed for both animal models in DD pigs. The observed heritability estimate of LMA in LL in this study was similarly moderate but with far higher magnitude compared with that indicated by Son et al. [27] of 0.20, while Chen et al. [28] reported higher heritability for LMA of 0.49 in LL. The heritability estimates of LMA in YY pigs were close with those observed by Johnson et al. [15] of 0.24 (loin eye area) in LW and Son et al. [27] of 0.25 in YY, but far lower than that reported by Cai et al. [26] of 0.57 for the same pig breed. Moreover, the observed heritability of LMA in DD in this study was found to be lower than those observed by Hoque et al. [22] of 0.45 (eye muscle area), Son et al. [27] of 0.22, and Lopez et al. [29] of 0.24 in DD pigs.

The heritability estimates for LP in this study were moderate to high in all breeds ranging from 0.22 to 0.42. An identical heritability for LP was observed for both animal models 1 and 2 in DD and LL, while model 1 had the highest heritability estimates in YY. Among three purebred pigs, the highest heritability for LP was observed in models 1 (0.42) and 2 of DD (0.42), and model 1 (0.42) of YY pigs, while an intermediate estimate was observed in model 2 (0.26) of YY, and the lowest heritability was observed in models 1 and 2 in LL pigs with an identical heritability of 0.22. The noticeable big difference in the heritability of LP between models 1 and 2 in YY could be attributed to a considerable fraction (9%) of the common pen effect on this particular trait. The observed high heritability for LP in model 1 of YY pigs was in agreement with the findings of Son et al. [27] of 0.46 in YY, Gilbert et al. [9] of 0.53 in LW females and castrated males, Saintilan et al. [8] of 0.55 in the LW sire breed and 0.60 in the LW dam breed, and Labroue et al. [21] of 0.76 in LW pigs. However, these references did not include the common pen effect and therefore do not assess the pen effect to the heritability of LP in YY as similar to animal model 2 in this study. Meanwhile, the observed heritability for LP in DD was similarly high with those observed by Son et al. [27], Lopez et al. [29], Gjerlaug-Enger et al. [30], and Cabling et al. [31] of 0.39, 0.42, 0.57, and 0.73, respectively. Finally, the observed heritability of LP in LL was far lower than those observed by Labroue et al. [21], Gjerlaug-Enger et al. [30], Saintilan et al. [8], and Son et al. [27] with estimates ranging from 0.49 to 0.66, respectively.

In general, the observed differences in the heritability estimates of production traits can be attributed to the differences in the number and sex of tested animals, differences in fixed effects, fixed covariates and the random effect included in the animal model for the estimation of genetic parameters, variation in testing duration, testing age and weight, and differences in measurement methods for carcass compositions. Given these differences, the majority of production traits of the three pig breeds used in this study had moderate to high heritability, indicating a fast improvement for these traits once they are considered as part of the breeding program.

### 3.5. Additive Common Spatial Pen Effect

The common spatial pen effect explained a varied percentage variability for feed efficiency and production traits in three purebred pigs in this study. Compared between LL and YY which have similar animal models, the earlier breed had slightly higher percentage variance due to the common spatial pen effect for FCR, RFI1, RFI2, RFI3, RFI4, and RFI5 (Table 5, Table 6 and Table 7). Meanwhile, a low percentage pen variance due to the common spatial pen effect was observed in most production traits of LL and YY except only for a considerable proportion observed in the LP of the latter breed (Table 8). For DD, which have animal models different than earlier breeds, a substantial percentage of variance explained by the common spatial pen effect was observed in all feed efficiency traits, while a smaller proportion was observed in all production traits.

The common spatial pen effect accounted for about 10% to 19% and 7% to 10% of the total variability for feed efficiency traits in LL and YY, respectively. In LL, the common spatial pen effect explained zero percentage to the total variability of BFT, LMA, and LP, while it explained 6% to the total variability of ADG. Furthermore, in YY pigs, the common spatial pen effect explained zero percentage to the total variability of ADG and BFT, while it explained 3% and 9% to the total variability of LMA and LP, respectively. In DD pigs, the percentage of variance due to common spatial pen effects ranged from 17% to 22% in all feed efficiency traits, while only 0% to 2% was observed in all production traits.

The observed percentage of variance due to the common spatial pen effect in feed efficiency traits of DD were slightly higher than those observed by Lu et al. [5] in G:F and six RFI values ranging from 14% to 17%, while a higher and comparable percentage value was observed by the same researchers in on-test ADG (18%) and BFT (2%), respectively. In the study of Do et al. [4], lower percentage variance due to common pen effect were observed for FCR (3% to 5%), RFI1 (3% to 5%), and RFI2 (2% to 4%), while reported a similarly low but with a slightly higher percentage value for ADG (3% to 5%) and BFT (3% to 6%) in three pig breed populations, similar to this study. However, the latter reference did not specify whether physical space used by different batches of pigs or a social pen effect shared by all pen mates per batch were included in their study.

Overall, the common spatial pen effect varies in every trait and breed used in this study. Despite such variability, most of the traits belonging to the feed efficiency category were observed to have a higher percentage of variance due to the common pen effect as compared to most traits measured during the end of the testing period such as BFT, LMA, and LP (except in YY) as also observed by Do et al. [4]. Therefore, the inclusion of the random common spatial pen effect might bring a substantial improvement in the estimation of genetic parameters, particularly on feed efficiency traits. However, checking the goodness of fit through log-likelihood, AIC, and BIC is important to consider to determine which model would provide the most precise estimates of the breeding value.

### 3.6. Model Comparison

The AIC scores for each feed efficiency (Table 5, Table 6 and Table 7) and production (Table 8) trait were used as the basis for model comparison. In DD, model 1 had 23.06 to 34.28 points lower AIC score than model 2 in all feed efficiency traits, while the opposite was observed in production traits where model 2 had a lower AIC score ranging from 0.40 to 1.02 points than model 1. An almost similar observation but with a lower AIC score difference was observed in YY where model 1 had 0.58 to 1.55 points lower AIC score for all feed efficiency traits, while model 2 had 0.74 to 1.00 points lower AIC score for most production traits except for LP which had a slightly lower AIC score in model 1. However, in LL pigs, model 1 had 1.37, 0.02, and 0.03 points lower AIC score than model 2 for FCR, RFI1, and RFI3, respectively, while model 2 had only 0.04, 0.05, and 0.13 lower AIC scores than model 1 for RFI2, RFI4, and RFI5, respectively. The same breed also recorded 0.84 to 1.01 points lower AIC score for all production traits when using model 2.

Based on the set cut-off threshold value of ≤2 [13] and ≤6 [14] as described earlier, the model with only the random animal and common litter effect in DD had the best model fit for feed efficiency traits, while for production traits the model with the random animal, common litter, and common spatial pen effect had the lowest AIC scores but not greater than the set cut-off threshold value (≤2), indicating that both models have no significant difference with each other. Similarly, both feed efficiency and production traits of YY and LL had also a smaller AIC score difference than the set cut-off threshold value (≤2), indicating that models with or without the common pen effect for both breeds would also be considered not different from each other. According to Hsu et al. [32], the litter effects and either pen or pen mate permanent environmental effects must be included when performing a genetic evaluation for ADG, BFT, and LMA of growing swine. Therefore, despite a small fraction of the pen effect and a smaller AIC score difference in production traits, the inclusion of the litter and pen effect should be considered for traits commonly measured towards the end of the test (BFT, LMA, and LP).

### 3.7. Correlation between Feed Efficiency and Production Traits in Duroc Pigs

The genetic and phenotypic correlation between feed efficiency and production traits in DD is presented in Table 9. Specifically, the genetic and phenotypic correlations of FCR to ADG, BFT, LMA, and LP for this breed were −0.18 and −0.19, 0.52 and 0.20, −0.08 and −0.05, −0.78 and −0.20, and 0.53 and 0.45, respectively. These observations were in a similar direction to those observed by Hoque et al. [20] who reported a negative moderate genetic and low phenotypic correlation between FCR and ADG (−0.26 and −0.32) and a positive high genetic and low phenotypic correlation between FCR and BFT (0.61 and 0.24). Hoque et al. [22] also reported a high positive and moderate negative genetic correlation between FCR and BFT (0.52) and between FCR and EMA (−0.49), respectively.

The genetic and phenotypic correlation of five measures of RFI to FCR and ADG was close to one (0.91 to 0.94 and 0.93 to 0.94) and not different to zero (0.05 to 0.06 and 0.00), respectively. The RFI measures in DD pigs with the highest genetic and phenotypic relationship to FCR were RFI1 (0.94) and RFI3 (0.94). Such a high genetic and phenotypic correlation between RFI without BFT as a covariate (RFI1 and RFI3) and FCR was in agreement with those observed by Hoque et al. [22] and Do et al. [4] in DD with an identical genetic correlation of 0.85 and a phenotypic correlation ranging from 0.84 to 0.88. The latter researchers [4] also reported a not different to zero and close to zero genetic and phenotypic correlation between RFI1 and ADG (0.16 and 0.01), and between RFI2 and ADG (0.04 to −0.01) for the same pig breed. These two measures of RFI used by Do et al. [4] have almost similar covariates in RFI1 and RFI2 in this study, except that the CG and ITA were not included by the said researchers. The measures of RFI without BFT as a covariate in this study had a different level of genetic and phenotypic correlation to remaining production traits. Specifically, RFI1 and RFI3 had a high and positive genetic (0.51) and low phenotypic (0.20) correlation to BFT, a close to zero genetic (−0.14 and −0.16) and not different to zero phenotypic correlation (−0.04 and 0.00) to LMA, a high and negative genetic (−0.77 to −0.78) and low phenotypic (−0.20 and −0.21) correlation to LP, and a high and positive genetic (0.70 to 0.71) and phenotypic correlation (0.63) to total feed intake (FI), respectively. Some of these observations were in a similar direction to those observed by Hoque et al. [16] in the RFI model without BFT (RFI1 and RFI3) as a covariate for the same pig breed. Particularly, these researchers reported that RFI measures without BFT as a covariate had a high and positive genetic correlation to BFT (0.76 and 0.77) and daily feed intake (0.77 and 0.78), and a moderate to high and negative genetic correlation between the same RFI measures and loin eye area (−0.46 and −0.60).

Meanwhile, compared with earlier discussed measures of RFI, the remaining measures of RFI calculated with BFT as a covariate (RFI2, RFI4, and RFI5) had a lower genetic and phenotypic correlation for the most production traits. Specifically, the RFI2, RFI4, and RFI5 showed a low and positive genetic correlation (0.21 to 0.22) and a not different to zero phenotypic correlation (0.01) to BFT. Similar RFI measures had a high negative genetic (−0.57 and −0.58) and an identical close to zero phenotypic correlation (−0.08) to LP, and also had an identical high and positive genetic (0.67) and phenotypic correlation (0.61) to FI. However, RFI2, RFI4, and RFI5 had a higher genetic correlation to LMA (−0.23 to −0.25) as compared to those observed in RFI1 and RFI3. Hoque et al. [16] also observed that RFI with BFT as a covariate (RFI2 and RFI4) had a similar direction but with a lower genetic correlation (0.07 and 0.11) and comparable phenotypic (0.00 and 0.01) correlation to BFT, while both RFI measures had moderate genetic (0.56 and 0.58) and phenotypic (0.56 and 0.57) correlation to daily feed intake (DFI). Although total feed intake (FI) and not DFI were correlated to RFI measures in this study, the same impact on the amount of feed intake is expected once these RFI measures are considered as part of the breeding program.

Generally, these observations suggest that selection for DD with low RFI would result in the reduction in feed intake without adversely affecting daily weight gain. Specifically, selection for a lower RFI with no BFT as a covariate (RFI1 and RFI3) would result in the reduction in feed intake, favorable decrease in BFT, and increase in meat LP on a stronger scale than RFI with BFT as a covariate (RFI2, RFI4, and RFI5). Thus, with these observations and a comparable genetic correlation of RFI measures with and without BFT as a covariate to production traits and feed intake, the selection for low RFI1 appears to be the most advantageous among RFI measures in DD pigs due to its slightly higher heritability, less model complexity, and applicability when records of BFT and LMA are not available in the data.

### 3.8. Correlation between Feed Efficiency and Production Traits in Landrace Pigs

The genetic and phenotypic correlation between feed efficiency and production traits in LL pigs is shown in Table 10. Particularly, the genetic and phenotypic correlation of FCR to ADG (−0.32 and −0.21) was higher than the rest of the feed efficiency traits. This was close to the genetic correlation between the same traits reported by Hoque et al. [20] and Do et al. [4] of −0.35 and −0.31 for the same pig breed, respectively. Compared to measures of RFI with BFT as a covariate (RFI2, RFI4, and RFI5), the RFI without BFT as a covariate (RFI1 and RFI3) also showed a higher genetic and phenotypic correlation to FCR in this study with an identical genetic and phenotypic correlation coefficient of 0.96. Do et al. [4] also reported the same findings for the same breed where measures of RFI without BFT as a covariate had a higher genetic (0.91) and phenotypic (0.95) correlation to FCR. Apart from having a strong relationship with each other, the FCR, RFI1, and RFI3 had also a close genetic and phenotypic correlation for most production traits in this study, except for the moderate genetic and phenotypic relationship observed between FCR and ADG as described earlier, and a lower genetic and phenotypic correlation between FCR and FI (0.30 and 0.42). Specifically, the FCR, RFI1, and RFI3 had a close moderate genetic (0.36 to 0.45) and phenotypic (0.36 to 0.39) correlation to BFT, low to moderate negative genetic (−0.18 to −0.21) and zero phenotypic (−0.01 to 0.00) correlation to LMA, and high negative genetic (−0.75 to −0.83) and moderate negative phenotypic (−0.34 to −0.35) correlation to LP, respectively.

Compared to those observed in RFI1 and RFI3, the RFI measures with BFT (RFI2, RFI4, and RFI5) as a covariate in LL had a lower genetic correlation to FCR, BFT, LP, and FI, except for ADG which had a stronger genetic correlation to latter RFI measures, and LMA which had an almost equal genetic and phenotypic correlation to all RFI measures in this study. Explicitly, the RFI2, RFI4, and RFI5 had a high and positive genetic (0.90) and phenotypic (0.89) correlation to FCR, a not different to zero genetic (−0.04) and phenotypic (0.00) correlation to BFT, a moderate and negative genetic correlation (−0.45 to −0.46) and close to zero phenotypic correlation (−0.13 to −0.14) to LP, a moderate and positive genetic (0.34 to 0.35) and high and positive phenotypic correlation (0.54, identical for three RFI measures) to FI, respectively. The observed lower genetic correlation of RFI measures with BFT as a covariate than RFI measures without BFT as a covariate to BFT was in agreement with those reported by Hoque et al. [20] of 0.06 and Do et al. [4] of −0.08 in LL, and Mrode and Kennedy [19] of 0.15 in DD, LL, and YY boars.

However, despite the higher correlation of FCR to RFI1 and RFI3, a weaker genetic and phenotypic correlation between RFI1 and RFI3 to ADG compared to FCR was still observed in this study with an identical genetic and phenotypic correlation coefficient of −0.11 and −0.01, respectively. These observations were lower than the genetic correlation of RFI2, RFI4, and RFI5 to ADG having an identical correlation coefficient of −0.19. The observed stronger genetic correlation between RFI measures with BFT as a covariate and ADG in this study was in agreement but with an opposite direction and larger difference than those obtained by Hoque et al. [20] and Mrode and Kennedy [19], where RFI measures with BFT as a covariate had only a 0.02 and 0.03 higher point difference against RFI measures with only ADG as a covariate.

Overall, these observations suggest that selection against all RFI measures in LL pigs would reduce feed intake without adversely affecting the daily weight gain. Specifically, selection for lower measures of RFI without BFT as a covariate (RFI1 and RFI3) would reduce FI, favorably decrease BFT, and increase LP on a stronger scale compared to RFI with BFT as a covariate (RFI2, RFI4, and RFI5). Similar to DD pigs, selection for a less complex RFI1 might be better to include in the selection criteria for improving feed efficiency, reducing BFT, and increasing LP in LL pigs.

### 3.9. Correlation between Feed Efficiency and Production Traits in Yorkshire Pigs

The genetic and phenotypic correlation between feed efficiency and production traits in YY is presented in Table 11. Similar to DD and LL, the RFI measures in YY which had the highest genetic and phenotypic correlation to FCR were RFI1 (0.92 and 0.95) and RFI3 (0.94 and 0.93). However, despite the high correlation of RFI1 and RFI3 to FCR, differences in their genetic and phenotypic correlation with the majority of production traits and FI were still noticeable. Specifically, the RFI1 and RFI3 had a higher genetic correlation of 0.51 and 0.55 to ADG, 0.38 and 0.39 to BFT, −0.20 and −0.21 to LP, and 0.81 and 0.84 to FI compared to those observed in FCR, respectively. Meanwhile, the FCR had an unfavorable contrasting genetic and phenotypic correlation (0.33 and −0.23) to ADG, and a higher genetic correlation to LMA (0.80) compared to RFI1 and RFI3. This observed contrasting correlation between FCR and ADG in YY pigs was also presented in the study of Do et al. [4] with a corresponding genetic and phenotypic correlation of 0.26 and −0.12, respectively. Apart from ADG, the earlier observed higher genetic correlation between BFT and RFI measures without BFT as a covariate than between FCR and BFT was supported by Mrode and Kennedy [19], Johnson et al. [15], and Do et al. [4]. Moreover, the observed unfavorable genetic correlation of RFI1 to ADG in this study was stronger but with a similar direction with those observed by Do et al. [4] between RFI1 (with only IBW and ADG as a covariate) and ADG of 0.25, while the genetic correlation reported by the same researchers between the same RFI measure (RFI1) and BFT of 0.43 was comparable with that observed in this study.

On the other hand, the RFI measures with BFT as a covariate (RFI2, RFI4, and RFI5) had also a high and positive genetic (0.91, identical for three RFI measures) and phenotypic (0.89 to 0.90) correlation to FCR, a not different to zero genetic (0.05 to 0.07) and zero phenotypic correlation to ADG, a low and negative genetic (−0.26 to −0.28) and not different to zero phenotypic (−0.01) correlation to BFT, a high and positive genetic (0.55 to 0.57) and not different to zero phenotypic (0.01 to 0.05) correlation to LMA, a low and positive genetic (0.21 to 0.22) and not different to zero phenotypic (−0.03 to −0.04) correlation to LP, and a moderate positive genetic (0.43 to 0.44) and phenotypic (0.53 to 0.54) correlation to FI, respectively. The observed negative genetic correlation of RFI measures with BFT (RFI2 and RFI4) as a covariate and the RFI measure with BFT and metabolic body weight (MBW) as covariates (RFI5) to BFT were in agreement but with a slightly higher correlation coefficient with those observed by Nguyen et al. [24] in LW pigs with a corresponding genetic correlation of −0.20 and −0.16, respectively.

Furthermore, the close to zero genetic and phenotypic correlation of RFI2 and RFI4 to ADG was in agreement but with a slightly lower genetic correlation than those observed by Johnson et al. [15] in LW boars of 0.17 and 0.18 between ADG and RFI measures with similar covariates to RFI2 and RFI4 in this study. However, similar researchers observed that RFI measures with BFT as a covariate had an opposite genetic and phenotypic correlation to BFT (0.20 and 0.22) and LEA (−0.31, identical in both RFI measures) compared with those observed in this study. Gilbert et al. [9] also reported opposing findings where the RFI measure with ADG and BFT as covariates had a favorable moderate negative genetic correlation to ADG of −0.35 in LW pigs.

Overall in YY, the observed genetic correlation among feed efficiency and production traits indicates that selection against FCR, RFI1, and RFI3 decreases feed intake but would also bring a negative impact to daily weight gain and a disadvantageous change to LMA in YY pigs. Meanwhile, selection for a lower RFI with BFT as a covariate (RFI2, RFI4, and RFI5) would reduce feed intake without adversely affecting daily gain but would likely bring disadvantageous change in BFT, LMA, and LP. Given the obvious comparable genetic correlation of RFI with BFT as a covariate to production traits and feed intake, the selection against less complex RFI2 among this RFI category is the most advantageous RFI measure in YY pigs.

## 4. Conclusions

The FCR had moderate heritability in all breeds, while the majority of RFI measures in DD and YY and all RFI measures in LL pigs had low and moderate heritability, respectively. These observations indicate a slow to moderate response of RFI measures to selection. The genetic correlation of all RFI measures to production traits and total feed intake revealed that selection against RFI2, RFI4, and RFI5 would likely result in the reduction in feed intake without affecting ADG in all breeds; advantageous change to BFT in DD, and LP in DD and LL; and disadvantageous change in BFT, LMA, and LP in YY. Meanwhile, selection against RFI1 and RFI3 in DD and LL would likely result in the reduction in FI and advantageous change in BFT and LP on a stronger scale compared to RFI2, RFI4, and RFI5. Among RFI measures, the RFI1 was found to be the most advantageous and less complex RFI measure in DD and LL, while RFI2 was the most preferred RFI measure in YY pigs. However, RFI measures with BFT as a covariate could still be applied for DD and LL when weaker genetic correlations to BFT and LP are desired and if carcass components records are available. Moreover, the inclusion of the common spatial pen effect in the animal model brings a substantial and small fraction to the total variability in most feed efficiency and production traits in three pig breeds, respectively. The obtained genetic parameters and correlation coefficients for breed-specific RFI measures in this study can be used in future studies which aim to improve the feed efficiency of the swine industry.

## Figures and Tables

**Table 1:** Number of pigs and breed from two breeding farms.

Farm	Duroc	Landrace	Yorkshire
A	ND	131	188
B	603	164	153
Total	603	295	341

ND, no data available.

**Table 2:** List of fixed effects and covariates included in estimating the genetic parameters for each trait of three purebred pigs.

Traits	Fixed Effects			Fixed Covariates			
	Batch	Farm-Batch	Test Day	ITA	IBW	FTA	FBW
RFI1	D	L,Y	NS	-	-	-	-
RFI2	D	L,Y	NS	-	-	-	-
RFI3	D	L,Y	NS	-	-	-	-
RFI4	D	L,Y	NS	-	-	-	-
RFI5	D	L,Y	NS	-	-	-	-
FCR	D	L,Y	NS	NS	D,L,Y	-	-
ADG	D	L,Y	NS	NS	D,L,Y	-	-
BFT	D	L,Y	NS	-	-	NS	D,L,Y
LMA	D	L,Y	NS	-	-	NS	D,L,Y
LP	D	L,Y	NS	-	-	NS	D,L,Y

D, Duroc; L, Landrace; Y, Yorkshire; NS, not significant; RFI1-RFI model 1; RFI2-RFI model 2; RFI3-RFI model 3; RFI4-RFI model 4; RFI5–RFI model 5; ADG, on-test average daily gain; FCR, feed conversion ratio; BFT, ultrasound backfat thickness; LMA, ultrasound loin muscle area; LP; lean percentage; Batch and Farm-Batch, fixed effects of testing batch or animals tested at the same time and location; ITA, initial testing age; IBW; initial testing weight; FTA; final testing age; FBW, final testing weight.

**Table 3:** Partial regression estimates of daily feed intake on initial testing age and weight, production, and maintenance traits of Duroc, Landrace, and Yorkshire Pigs.

Breed	RFI	ITA	IBW	ADG	BFT	LMA	AMBW	RMSE	R^2^	AIC
DD	1	−0.01 ± 0.005	0.01 ± 0.002	2.04 ± 0.07	-	-	-	0.1712	0.7628	−1496.29
	2	−0.006 ± 0.005	0.01 ± 0.002	1.83 ± 0.08	0.019 ± 0.004	-	-	0.1681	0.7717	−1517.35
	3	−0.01 ± 0.005	0.01 ± 0.002	2.02 ± 0.07	-	−0.0025 ± 0.003	-	0.1712	0.7632	−1495.21
	4	−0.006 ± 0.005	0.01 ± 0.002	1.82 ± 0.08	0.019 ± 0.004	−0.0013 ± 0.003	-	0.1682	0.7718	−1515.61
	5	−0.006 ± 0.005	-	1.52 ± 0.10	0.019 ± 0.004	−0.0013 ± 0.003	0.096 ± 0.02	0.1683	0.7717	−1515.32
LL	1	0.006 ± 0.005	0.011 ± 0.003	2.09 ± 0.10	-	-	-	0.1441	0.8047	−809.43
	2	0.004 ± 0.005	0.009 ± 0.003	1.61 ± 0.11	0.037 ± 0.005	-	-	0.1326	0.8354	−857.98
	3	0.006 ± 0.005	0.011 ± 0.003	2.08 ± 0.10	-	−0.0005 ± 0.004	-	0.1444	0.8047	−807.45
	4	0.004 ± 0.005	0.009 ± 0.003	1.62 ± 0.11	0.037 ± 0.005	0.0005 ± 0.004	-	0.1329	0.8354	−856.00
	5	0.004 ± 0.005	-	1.33 ± 0.15	0.037 ± 0.005	0.0006 ± 0.004	0.083 ± 0.02	0.1329	0.8353	−855.83
YY	1	−0.002 ± 0.004	0.012 ± 0.003	1.99 ± 0.08	-	-	-	0.1287	0.7983	−1020.44
	2	−0.003 ± 0.004	0.011 ± 0.002	1.58 ± 0.10	0.033 ± 0.005	-	-	0.1207	0.8230	−1063.07
	3	−0.002 ± 0.004	0.012 ± 0.003	2.00 ± 0.08	-	0.002 ± 0.003	-	0.1288	0.7986	−1018.88
	4	−0.004 ± 0.004	0.011 ± 0.002	1.59 ± 0.10	0.033 ± 0.005	0.0019 ± 0.003		0.1209	0.8233	−1061.51
	5	−0.004 ± 0.004	-	1.28 ± 0.14	0.033 ± 0.005	0.0017 ± 0.003	0.088 ± 0.02	0.1214	0.8217	−1058.57

ITA, initial testing age; IBW, initial testing weight; ADG, on-test average daily gain; BFT, backfat thickness; LMA, loin muscle area; AMBW, average metabolic body weight; RMSE, root mean square error; R^2^, coefficient of determination; AIC, Akaike information criterion.

**Table 4:** Descriptive statistics (mean ± SD) of production and feed efficiency traits for Duroc, Landrace, and Yorkshire pigs.

Traits	Unit	Duroc		Landrace		Yorkshire	
		Mean	SD	Mean	SD	Mean	SD
ADFI	kg/day	2.48	0.34	2.14	0.30	2.11	0.27
FI	kg	152.79	21.30	144.63	21.59	145.80	21.23
ADG	g/day	1067.32	129.55	905.63	122.93	900	120.00
BFT	mm	14.23	2.26	11.96	2.29	11.73	2.14
AMBW	kg	13.43	0.74	12.40	0.75	12.32	0.85
FCR	kg/kg	2.30	0.19	2.37	0.19	2.33	0.19
LMA	cm^2^	26.03	2.98	27.89	2.53	28.31	2.52
LP	%	56.95	2.23	58.24	2.92	58.91	2.58
RFI1	g/day	0.00	167.78	0.00	134.55	0.00	121.70
RFI2	g/day	0.00	164.59	0.00	123.50	0.00	113.99
RFI3	g/day	0.00	167.65	0.00	134.55	0.00	121.62
RFI4	g/day	0.00	164.56	0.00	123.50	0.00	113.92
RFI5	g/day	0.00	164.60	0.00	123.53	0.00	114.41

ADFI, average daily feed intake; FI, total feed intake; ADG, on-test average daily gain; BFT, backfat thickness; AMBW, average metabolic body weight; FCR, feed conversion ratio; LMA, loin muscle area; LP, lean percentage; RFI1-RFI model 1; RFI2-RFI model 2; RFI3-RFI model 3; RFI4-RFI model 4; RFI5-RFI model 5.

**Table 5:** Heritability estimates and (co)variance components for feed efficiency and production traits in Duroc pigs using two different models.

Traits	Model 1					Model 2						Diff
	$\sigma_{a}^{2}$	$\sigma_{e}^{2}$	h^2^	c^2^	AIC	$\sigma_{a}^{2}$	$\sigma_{e}^{2}$	h^2^	c^2^	p^2^	AIC	
FCR	7.6 × 10^−3^	1.7 × 10^−2^	0.28 ± 0.12	0.10	733.95	6.3 × 10^−3^	1.5 × 10^−2^	0.22 ± 0.10	0.11	0.17	757.01	23.06
RFI1	6808.47	19,789.00	0.23 ± 0.11	0.12	−3298.33	5633.53	16,177.20	0.17 ± 0.09	0.12	0.20	−3267.09	31.24
RFI2	5498.67	20,197.40	0.19 ± 0.10	0.11	−3289.50	4594.60	16,431.10	0.15 ± 0.09	0.11	0.22	−3255.40	34.10
RFI3	6748.99	19,734.60	0.22 ± 0.11	0.12	−3297.75	5526.62	16,128.00	0.17 ± 0.09	0.13	0.20	−3266.24	31.51
RFI4	5463.47	20,163.20	0.19 ± 0.10	0.11	−3289.29	4535.08	16,399.80	0.15 ± 0.09	0.11	0.22	−3255.06	34.23
RFI5	5422.38	20,207.90	0.19 ± 0.10	0.11	−3289.50	4499.71	16,437.60	0.15 ± 0.09	0.11	0.22	−3255.22	34.28

$\sigma_{a}^{2}$, additive genetic variance; $\sigma_{e}^{2}$, residual variance; h^2^, heritability estimates; c^2^, variance ratio for common litter effect; p^2^, variance ratio for spatial common pen effect; AIC, Akaike information criterion; Diff, difference (absolute) between 2 AIC score; FCR, feed conversion ratio; RFI1-RFI model 1; RFI2-RFI model 2; RFI3-RFI model 3; RFI4-RFI model 4; RFI5-RFI model 5.

**Table 6:** Heritability estimates and (co)variance components for feed efficiency and production traits of Landrace pigs using two different models.

Traits	Model 1				Model 2					Diff
	$\sigma_{a}^{2}$	$\sigma_{e}^{2}$	h^2^	AIC	$\sigma_{a}^{2}$	$\sigma_{e}^{2}$	h^2^	p^2^	AIC	
FCR	1.1 × 10^−2^	1.7 × 10^−2^	0.39 ± 0.18	296.18	1.1 × 10^−2^	1.7 × 10^−2^	0.31 ± 0.16	0.19	297.55	1.37
RFI1	7241.21	13,991.10	0.34 ± 0.16	−1451.43	7190.00	13,641.40	0.31 ± 0.16	0.10	−1451.41	0.02
RFI2	5954.04	11,903.90	0.33 ± 0.16	−1429.24	5930.29	11,599.10	0.30 ± 0.15	0.10	−1429.28	0.04
RFI3	7226.12	14,004.50	0.34 ± 0.17	−1451.44	7165.44	13,662.10	0.31 ± 0.16	0.10	−1451.41	0.03
RFI4	5966.16	11,890.20	0.33 ± 0.16	−1429.21	5950.92	11,579.80	0.31 ± 0.15	0.10	−1429.26	0.05
RFI5	6011.96	11,866.20	0.34 ± 0.16	−1429.30	6001.89	11,569.00	0.31 ± 0.16	0.10	−1429.43	0.13

$\sigma_{a}^{2}$, additive genetic variance; $\sigma_{e}^{2}$, residual variance; h^2^, heritability estimates; c^2^, variance ratio for common litter effect; p^2^, variance ratio for spatial common pen effect; AIC, Akaike information criterion; Diff, difference (absolute) between 2 AIC score; FCR, feed conversion ratio; RFI1-RFI model 1; RFI2-RFI model 2; RFI3-RFI model 3; RFI4-RFI model 4; RFI5-RFI model 5.

**Table 7:** Heritability estimates and (co)variance components for feed efficiency and production traits of Yorkshire pigs using two different models.

Traits	Model 1				Model 2					Diff
	$\sigma_{a}^{2}$	$\sigma_{e}^{2}$	h^2^	AIC	$\sigma_{a}^{2}$	$\sigma_{e}^{2}$	h^2^	p^2^	AIC	
FCR	5.5 × 10^−3^	1.9 × 10^−2^	0.22 ± 0.16	373.88	4.5 × 10^−3^	1.9 × 10^−2^	0.17 ± 0.14	0.10	375.43	1.55
RFI1	3128.17	13,627.50	0.19 ± 0.15	−1682.86	2713.26	13,572.70	0.15 ± 0.14	0.07	−1682.20	0.66
RFI2	2931.72	11,785.00	0.20 ± 0.15	−1662.59	2557.00	11,740.50	0.17 ± 0.14	0.08	−1661.96	0.63
RFI3	2917.86	13,791.30	0.18 ± 0.15	−1682.77	2534.21	13,716.80	0.14 ± 0.14	0.07	−1682.13	0.64
RFI4	2769.89	11,908.30	0.19 ± 0.15	−1662.48	2430.44	11,843.70	0.16 ± 0.14	0.08	−1661.90	0.58
RFI5	2847.00	11,967.40	0.19 ± 0.15	−1663.80	2504.57	11,901.40	0.16 ± 0.14	0.08	−1663.22	0.58

$\sigma_{a}^{2}$, additive genetic variance; $\sigma_{e}^{2}$, residual variance; h^2^, heritability estimates; c^2^, variance ratio for common litter effect; p^2^, variance ratio for spatial common pen effect; AIC, Akaike information criterion; Diff, difference (absolute) between 2 AIC score; FCR, feed conversion ratio; RFI1-RFI model 1; RFI2-RFI model 2; RFI3-RFI model 3; RFI4-RFI model 4; RFI5-RFI model 5.

**Table 8:** Heritability estimates and (co)variance components for production traits in Duroc, Landrace, and Yorkshire pigs using two different models.

Breed	Traits	Model 1					Model 2						Diff
		$\sigma_{a}^{2}$	$\sigma_{e}^{2}$	h^2^	c^2^	AIC	$\sigma_{a}^{2}$	$\sigma_{e}^{2}$	h^2^	c^2^	p^2^	AIC	
DD	ADG	1882.76	8789.19	0.17 ± 0.10	0.05	−3026.23	1812.16	8708.76	0.16 ± 0.10	0.06	0.01	−3026.84	0.61
	BFT	2.42	1.01	0.68 ± 0.14	0.03	−632.86	2.48	0.96	0.69 ± 0.13	0.03	0.02	−633.12	0.26
	LMA	0.78	5.75	0.11 ± 0.09	0.07	−890.21	0.79	5.76	0.11 ± 0.09	0.07	0.00	−891.23	1.02
	LP	1.64	2.05	0.42 ± 0.13	0.07	−695.74	1.68	2.02	0.42 ± 0.13	0.06	0.01	−696.14	0.40
LL	ADG	3474.31	5703.07	0.38 ± 0.17	-	−1340.70	3355.90	5723.95	0.35 ± 0.17	-	0.06	−1341.54	0.84
	BFT	1.43	1.18	0.55 ± 0.18	-	−282.33	1.43	1.18	0.55 ± 0.18	-	0.00	−283.34	1.01
	LMA	2.07	3.38	0.38 ± 0.19	-	−383.20	2.08	3.38	0.38 ± 0.19	-	0.00	−384.20	1.00
	LP	1.16	4.04	0.22 ± 0.15	-	−381.52	1.16	4.04	0.22 ± 0.16	-	0.00	−382.52	1.00
YY	ADG	2757.26	5877.52	0.32 ± 0.17	-	−1575.93	2768.25	5868.95	0.32 ± 0.17	-	0.00	−1576.93	1.00
	BFT	1.47	0.69	0.68 ± 0.17	-	−292.77	1.47	0.70	0.68 ± 0.17	-	0.00	−293.78	1.01
	LMA	1.06	4.42	0.19 ± 0.13	-	−454.44	1.01	4.41	0.18 ± 0.13	-	0.03	−455.18	0.74
	LP	1.71	2.40	0.42 ± 0.18	-	−402.78	1.11	2.78	0.26 ± 0.16	-	0.09	−402.63	0.15

$\sigma_{a}^{2}$, additive genetic variance; $\sigma_{e}^{2}$, residual variance; h^2^, heritability estimates; c^2^, variance ratio for common litter effect; p^2^, variance ratio for spatial common pen effect; AIC, Akaike information criterion; Diff, difference (absolute) between 2 AIC score; ADG, on-test average daily gain; BFT, backfat thickness; LMA, loin muscle area; LP, lean percentage.

**Table 9:** Estimates of additive genetic correlation (r_g_) and phenotypic correlation (r_p_) between feeding efficiency traits with production traits and feed intake in Duroc boars.

Traits	FCR		RFI1		RFI2		RFI3		RFI4		RFI5	
	r_g_	r_p_	r_g_	r_p_	r_g_	r_p_	r_g_	r_p_	r_g_	r_p_	r_g_	r_p_
FCR	-	-	0.94	0.94	0.91	0.93	0.94	0.94	0.91	0.93	0.91	0.93
ADG	−0.18	−0.19	0.06	0.00	0.06	0.00	0.05	0.00	0.06	0.00	0.06	0.00
BFT	0.52	0.20	0.51	0.20	0.21	0.01	0.51	0.20	0.22	0.01	0.22	0.01
LMA	−0.08	−0.05	−0.16	−0.04	−0.25	−0.02	−0.14	0.00	−0.23	0.00	−0.23	0.00
LP	−0.78	−0.20	−0.77	−0.21	−0.57	−0.08	−0.78	−0.20	−0.58	−0.08	−0.58	−0.08
FI	0.53	0.45	0.71	0.63	0.67	0.61	0.70	0.63	0.67	0.61	0.67	0.61

FCR, feed conversion ratio; ADG, on-test average daily gain; BFT, backfat thickness; LMA, loin muscle area; LP, lean percentage; FI, total feed intake; RFI1-RFI model 1; RFI2-RFI model 2; RFI3-RFI model 3; RFI4-RFI model 4; RFI5-RFI model 5.

**Table 10:** Estimates of additive genetic correlation (r_g_) and phenotypic correlation (r_p_) between feeding efficiency traits with production traits and feed intake in Landrace pigs.

Traits	FCR		RFI1		RFI2		RFI3		RFI4		RFI5	
	r_g_	r_p_	r_g_	r_p_	r_g_	r_p_	r_g_	r_p_	r_g_	r_p_	r_g_	r_p_
FCR	-	-	0.96	0.96	0.90	0.89	0.96	0.96	0.90	0.89	0.90	0.89
ADG	−0.32	−0.21	−0.11	−0.01	−0.19	−0.01	−0.11	−0.01	−0.19	−0.01	−0.19	−0.01
BFT	0.36	0.36	0.45	0.39	−0.04	0.00	0.45	0.39	−0.04	0.00	−0.04	0.00
LMA	−0.21	−0.01	−0.19	−0.01	−0.18	0.00	−0.18	0.00	−0.19	−0.01	−0.18	−0.01
LP	−0.75	−0.34	−0.83	−0.35	−0.45	−0.13	−0.82	−0.35	−0.46	−0.14	−0.45	−0.13
FI	0.30	0.42	0.49	0.59	0.35	0.54	0.49	0.59	0.35	0.54	0.34	0.54

FCR, feed conversion ratio; ADG, on-test average daily gain; BFT, backfat thickness; LMA, loin muscle area; LP, lean percentage; FI, total feed intake; RFI1-RFI model 1; RFI2-RFI model 2; RFI3-RFI model 3; RFI4-RFI model 4; RFI5-RFI model 5.

**Table 11:** Estimates of additive genetic correlation (r_g_) and phenotypic correlation (r_p_) between feeding efficiency traits with production traits and feed intake in Yorkshire pigs.

Traits	FCR		RFI1		RFI2		RFI3		RFI4		RFI5	
	r_g_	r_p_	r_g_	r_p_	r_g_	r_p_	r_g_	r_p_	r_g_	r_p_	r_g_	r_p_
FCR	-	-	0.92	0.95	0.91	0.90	0.94	0.93	0.91	0.89	0.91	0.90
ADG	0.33	−0.23	0.51	0.01	0.05	0.00	0.55	0.01	0.07	0.00	0.06	−0.00
BFT	0.22	0.30	0.38	0.34	−0.26	−0.01	0.39	0.35	−0.27	−0.01	−0.28	−0.01
LMA	0.80	0.06	0.61	0.05	0.57	0.05	0.59	0.01	0.55	0.01	0.56	0.01
LP	−0.15	−0.17	−0.20	−0.21	0.22	−0.03	−0.21	−0.22	0.22	−0.04	0.21	−0.04
FI	0.67	0.36	0.81	0.59	0.43	0.54	0.84	0.59	0.44	0.54	0.43	0.53

FCR, feed conversion ratio; ADG, on-test average daily gain; BFT, backfat thickness; LMA, loin muscle area; LP, lean percentage; FI, total feed intake; RFI1-RFI model 1; RFI2-RFI model 2; RFI3-RFI model 3; RFI4-RFI model 4; RFI5-RFI model 5.

## Data Availability

Not applicable.

## Supplementary Materials

The following are available online at https://www.mdpi.com/article/10.3390/life11080830/s1, Table S1: Number of litters and animals in the pedigree records of each purebred pig.
