# Supplementary material for: Genetic Parameters for Different Measures of Feed Efficiency and Their Relationship to Production Traits in Three Purebred Pigs
Source: Life (Basel). 2021 Aug 13;11(8):830. doi: 10.3390/life11080830 (PMC8401224; doi:10.3390/life11080830)
Supplement: Supplementary file 1 [file life-11-00830-s001.zip › life-1321707-supplementary.pdf]

Table S1. Number of litters and animals in the pedigree records of each purebred pig.

Variable	Duroc	Landrace	Yorkshire
Litter	328	202	220
Pedigree	20,226	132,426	178,563
